# Unlocking the Potential of Vessel Density and the Foveal Avascular Zone in Optical Coherence Tomography Angiography as Biomarkers in Alzheimer’s Disease

**DOI:** 10.3390/healthcare12161589

**Published:** 2024-08-09

**Authors:** Iordanis Vagiakis, Christos Bakirtzis, Athina Andravizou, Demetrios Pirounides

**Affiliations:** 1 Department of Ophthalmology, AHEPA University Hospital, 54626 Thessaloniki, Greece; jvag_@outlook.com; 2 Second Department of Neurology, School of Medicine, Aristotle University of Thessaloniki, 54621 Thessaloniki, Greece; athina.andravizou9@hotmail.com

**Keywords:** OCT-A, Alzheimer’s disease, vessel density, foveal avascular zone

## Abstract

Alzheimer’s disease is the most prevalent form of dementia. Apart from its traditional clinical diagnostic methods, novel ocular imaging biomarkers have the potential to significantly enhance the diagnosis of Alzheimer’s disease. Ophthalmologists might be able to play a crucial role in this multidisciplinary approach, aiding in the early detection and diagnosis of Alzheimer’s disease through the use of advanced retinal imaging techniques. This systematic literature review the utilization of optical coherence tomography angiography biomarkers, specifically vessel density and the foveal avascular zone, for the diagnosis of Alzheimer’s disease. A comprehensive search was performed across multiple academic journal databases, including 11 relevant studies. The selected studies underwent thorough analysis to assess the potential of these optical coherence tomography angiography biomarkers as diagnostic tools for Alzheimer’s disease. The assessment of vessel density and the foveal avascular zone have emerged as a promising avenue for identifying and diagnosing Alzheimer’s disease. However, it is imperative to acknowledge that further targeted investigations are warranted to address the inherent limitations of the existing body of literature. These limitations encompass various factors such as modest sample sizes, heterogeneity among study populations, disparities in optical coherence tomography angiography imaging protocols, and inconsistencies in the reported findings. In order to establish the clinical utility and robustness of these biomarkers in Alzheimer’s disease diagnosis, future research endeavors should strive to overcome these limitations by implementing larger-scale studies characterized by standardized protocols and comprehensive assessments.

## 1. Introduction

Alzheimer’s disease (AD) is an incurable degenerative condition that impacts a person’s ability to perform daily tasks and interact socially due to its effects on cognitive function [1]. Currently, it is the most common neurodegenerative disease in the world [2]. As a result of aging populations and the global rising of life expectancy, the incidence of AD is anticipated to rise more in the coming years, particularly in developing countries. Moreover, the actual number of cases is likely to be substantially higher than reported due to underdiagnosis and limited access to healthcare in certain regions [3]. AD is defined by the presence of unique pathological alterations in the brain, namely the deposition of amyloid-beta protein (Aβ) and hyperphosphorylated tau proteins, leading to amyloid plaque and neurofibrillary tangle (NFT) formation, which cause local inflammation and degeneration of the brain ganglion cells [4,5,6]. The diagnosis of AD is primarily clinical [7]. Only a postmortem examination of brain tissue, specifically identifying the presence of neurofibrillary tangles and Aβ plaques in certain regions, can definitively confirm the diagnosis of AD [8]. In order to support the diagnosis of AD, identifying pathological proteins such as Aβ in cerebrospinal fluid, as well as utilizing sensitive and specific magnetic resonance imaging (MRI) findings, are often required. However, these methods may be invasive, costly, and time-consuming, making them less practical, especially in developing countries, as well as resulting in a significant disease burden that can be pretty costly [9,10]. Therefore, incorporating complementary diagnostic tools, especially noninvasive ones, could aid in the prompt identification of this disease [11]. The eye’s unique capability to provide noninvasive imaging of both neural tissue and microcirculation makes it an attractive area for biomarker research, especially since the retinal blood vessels and nerve fibers—highly visible components of the central nervous system—have been linked to various neurological conditions, including multiple sclerosis and stroke [12]. Moreover, several pathological changes can be observed in the different retina layers in patients with AD [13,14]. It is already well known and established that with the help of optical coherence tomography (OCT), a reduction is noted in the central retinal thickness, in the total retinal ganglion cells, and in the retinal nerve fiber layer of patients with AD [15,16,17,18]. Moreover, various studies have documented the morphological and functional changes in the cerebral blood vessels of individuals with AD [19,20]. It is proposed that those changes could be reflected in the retinal vasculature due to the common embryologic origins and anatomical and physiological characteristics of the retinal and cerebral microvasculature [21,22,23]. Therefore, ocular coherence tomography angiography (OCT-A), which focuses on the vascular complex of the retina, represents an efficient and promising imaging modality capable of detecting alterations in the vascular network of the ageing population, thus aiding in the diagnosis of neurodegenerative diseases [24]. Since its discovery in 2014 and its subsequent widespread application in common ocular disorders, OCT-A has been used as a potential biomarker for several pathologies [25]. The most prevalent retinal disorders, such as diabetic retinopathy and neovascular macular degeneration, have already established OCT-A biomarkers, which improve the accuracy of diagnosis, track disease progression, and enhance treatment approaches [26,27,28]. Moreover, OCT-A is proposed to be utilized in the diagnosis of various neurological conditions apart from AD [29,30,31]. Recently, the focus of the research has also been focused on the use of OCT-A in AD, which is promising and includes findings of enlargement in the foveal avascular zone (FAZ) and a reduction in the vessel density within the superficial and deep vascular complexes of the retina. The objective of this paper is to evaluate whether the foveal avascular zone (FAZ) and vessel density can serve as biomarkers for diagnosing Alzheimer’s disease (AD) and to suggest recommendations for future investigations of these potential biomarkers.

## 2. Materials and Methods

A comprehensive systematic search was conducted across multiple scholarly databases, including PubMed, Google Scholar, ScienceDirect, and Cochrane, utilizing relevant keywords such as “OCT-A in Alzheimer’s disease”, “OCT-A in dementia”, “Dementia biomarkers”, and “Alzheimer’s disease biomarkers”. The search included studies published until September 2023. The inclusion criteria for the selected studies were restricted to those that employed OCT-A in patients with confirmed AD, while studies focusing on individuals with mild cognitive impairment (MCI) were excluded. Additionally, studies that examined both AD and MCI patients were included. However, the analysis primarily focused on the changes observed in AD patients, particularly in relation to the FAZ and the superficial and deep vessel density. This rigorous search strategy aimed to identify the relevant literature and ensure the inclusion of studies specifically investigating OCT-A biomarkers in the context of AD. 

## 3. Results

Following the criteria outlined in the methods, 11 studies were selected and analyzed [32,33,34,35,36,37,38,39,40,41,42]. Thus, after providing a brief overview of the fundamental principles of OCT-A, this paper clarified the aspects regarding the changes in vessel density in the superficial and deep retinal layers, as well as the FAZ of AD patients, observed in these studies.

### 3.1. OCT-A Basic Principles

OCT-A, since its introduction in 2014, has been widely adopted as an imaging modality in ophthalmology, replacing conventional retinal vascular imaging modalities such as fluorescein angiography (FA) in many cases [43,44]. Compared to FA, it is noninvasive, eliminating all the possible side effects of intravenous contrast agent injections, and it is much safer and quicker [45]. In contrast to the two-dimensional images offered by FA, OCT-A enables the visualization of blood flow within specific retinal and choroidal layers, generating depth-resolved images of the retinal capillary plexuses [43]. Additionally, patients benefit from avoiding potential complications associated with intravenous fluorescein injections [46]. However, it should be noted that OCT-A is not a dynamic imaging modality and is, therefore, incapable of depicting leakages and other dynamic vascular phenomena [47]. The basic principles of OCT-A imaging acquisition are the same as conventional OCT [48,49]. Providing a more detailed explanation, OCT-A utilizes the analysis of decorrelation signals, which capture differences in the backscattered intensity or amplitude of OCT signals obtained from sequential OCT B-scans acquired at the exact cross-sectional location [47,50]. By comparing these signals, OCT-A generates a visual representation of blood flow patterns within the retina [51]. To ensure an accurate assessment of blood flow, any axial bulk motion resulting from patient movement is effectively mitigated [52]. Consequently, areas of motion detected between successive OCT B-scans primarily reflect the movement of erythrocytes specifically within retinal blood vessels [53,54]. Specific retinal layers are reproduced by segmenting the repeated B-scan images of the retinal vascular network [55]. In general, the retina is divided by various OCT-A software into different regions, as follows:

- The inner retina (from the ganglion cell layer to the inner plexiform layer);
- The middle retina (from the inner nuclear layer to the outer plexiform layer);
- The outer retina (from the outer nuclear layer to the external limiting membrane);
- The choriocapillaris;

Although several OCT-As are in production nowadays, the spectral domain OCT-As used in the studies examined in this review were manufactured by Optuvue (Fermont, CA, USA) and Zeiss (Jena, Germany) utilizing either the OptoVue AngioVue or the Zeiss AngioPlex software, respectively [56,57]. The segmentation scheme differs between them, creating slightly different boundaries for the specific retinal layers segmented [52,58]. More specifically, in OptoVue AngioVue software, the superficial plexus encompasses the region delimited by the posterior border of the inner plexiform layer (IPL) and the inner limiting membrane (ILM). Conversely, the deep plexus comprises the capillaries between the IPL’s posterior boundary and the outer plexiform layer (OPL). Finally, the choriocapillaris is defined as a distinct layer of capillaries found in a section measuring 30 μm in thickness and positioned immediately posterior to the retinal pigment epithelium (RPE) [59,60]. The segmentation strategy applied in the Zeiss AngioPlex system, as shown in Table 1, delineates the retinal structure into three distinct layers, namely the superficial, deep, and avascular retinal layers [61]. The superficial layer encompasses the upper 60% of the retinal depth, spanning from the inner limiting membrane (ILM) to a point approximately 110 μm above the retinal pigment epithelium (RPE), which coincides with the anterior extent of the inner plexiform layer (IPL). The deep retinal layer comprises the remaining 40% of the retinal depth, extending from the posterior border of the IPL to the anterior boundary of the outer nuclear layer (ONL). The avascular layer stretches from 110 μm above the RPE to the external limiting membrane (ELM) [58,62].

Each of these regions can be evaluated individually for the presence of pathological vessels, such as neovascularization, loss of capillary perfusion, and vessel tortuosity [28]. Moreover, the capillary perfusion density map, mean perfusion density, and vessel density can be calculated for superficial and deep-lying retinal vessels in the whole, superior, inferior, foveal, and parafoveal macular area, providing a grading system of the progressive alterations in blood vessel function [63]. The FAZ can also be examined and refers to the central area of the macula that lacks blood vessels and is enclosed by an uninterrupted network of capillary plexus. The preservation of FAZ integrity is crucial for maintaining normal visual acuity and, with the help of the OCT-A, can be quantitatively measured [64,65,66].

### 3.2. Changes in the Density of the Superficial Retinal Layer, Deep Retinal Layer, and the Foveal Avascular Zone

The details of the 11 studies regarding the patient sample size, the OCT-A used, the scan size, and the biomarkers examined are depicted in Table 2. In several studies, the vessel density measured in the superficial vascular complex of the parafoveal region was significantly lower in AD patients than in healthy controls [34,37,38,39]. On the other hand, other studies found no difference between the patients and the control groups [36,40]. Regarding the whole-enface vessel density, a significant decrease was observed in patients with AD compared to the control group [33,34,35,39]. Bulut et al. [33] using 6 × 6 macular scans with the Optovue OCT-A, found a significantly lower vessel density in the whole macular zone both foveally and parafoveally compared to the control group; however, in this study, the superficial and the deep vascular plexus were not investigated separately. In addition, Yoon et al. [34] found similar results regarding the vessel density and the perfusion density at the macula by dividing the scan area into 3 mm and 6 mm circles. Jing Wu et al. [37] located the decrease in vessel density only in a tiny and specific region of the scanned area in the superficial vascular complex in contrast to the deep vascular complex where the microvascular loss was profound almost in every other region, parafoveally and perifoveally. Chua et al. [38] using the Cirrus HD-5000 OCT-A (Carl Zeiss Meditec, Jena, Germany), scanned an area of 3 × 3 mm in the macula; however, in this study, only a tiny region with a radius of 2.5 mm from the fovea was examined, showing the decrease in vessel density in both superficial and deep complex. In the study of Xi Wang et al. [39] the vessel density in both the superficial and deep vascular complex was found to be decreased; however, after multivariate analysis and several adjustments, only the superficial vascular density was significantly lower compared to the control group.

**Figure 1:**
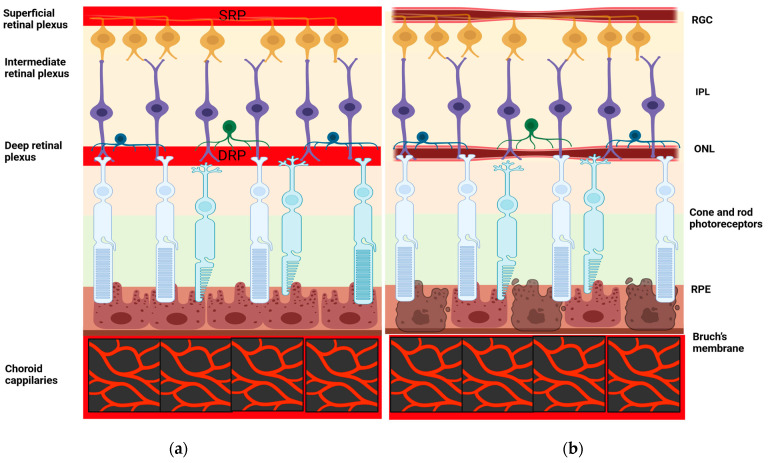
Retinal vessel densities in healthy individuals (**a**) and in individuals with Alzheimer’s disease (**b**). (**a**) In healthy individuals, the retina is supplied by two sets of vessels, the retinal (superficial, and deep plexus) and the choroidal capillaries. The superficial and deep plexuses are interconnected and vascularize the inner retina, while the outer retina is vascularized by the choroidal capillaries. (**b**) In patients with Alzheimer’s disease, the vessel density in the superficial and deep retinal plexuses is decreased. RPE is atrophic due to age. RGCs: retinal ganglion cells, IPL: inner plexiform layer (bipolar cells, horizontal cells and amacrine cells), ONL: outer nuclear layer (synaptic layer), RPE: retinal pigmented epithelium. Created with BioRender.com.

Regarding the vessel density in the deep vascular complex of the retina, a decrease in vessel density has been confirmed by several studies [36,37,38], while no significant difference was observed in other studies [35,39,40]. Wu et al. [37] performed a microvascular analysis in all four quadrants in the parafoveal and perifoveal annular zones of both superficial and deep vascular plexuses, finding a more prominent reduction in the deep vascular plexus, indicating that the smaller vessels in the deep vascular complex are more susceptible to disease progression compared to the larger vessels in the superficial retinal capillary plexus. This suggests that, in response to the ischemic and hypoxic effects caused by vasoconstriction and reduced blood flow in AD patients, the blood vessels in the deep vascular complex undergo earlier and more pronounced capillary shrinkage and tortuosity than those in the superficial vascular complex. Moreover, an increase in the density differences from the central to outer annular zones in the deep vascular complex was observed, which is likely linked to the disease progression. On the other hand, although Lahme et al. [35] found a decreased vessel density in the superficial layer between AD patients and controls, no significant difference was observed in the deep vascular complex and the FAZ. Notably, Zabel et al. [36] found the area with the most significant variation in vessel density in the deep vascular complex between the AD group and the control to be in the perifoveal region. According to Xie et al. [40] who used deep learning models to segment the OCT-A images in their study, although participants with AD exhibited impaired microvascular morphology and decreased vascular densities in both the superficial and deep vascular complex, the difference was not statistically significant. Querques et al. [41] found a non-significant minor reduction in perfusion density within the deep vascular plexus in individuals with AD, indicating that functional abnormalities in retinal vessels may precede any morphological changes. Retinal vessel densities under normal conditions and in Alzheimer’s disease are presented in Figure 1.

Regarding the FAZ, several autopsy studies have revealed the presence of amyloid deposition in cerebral capillaries, leading to a reduction in the number of capillaries in the brain [67,68]. Given the anatomic and physiologic similarities between the cerebral and retinal vessels, the accumulation of amyloids might also happen in the retina, leading to the damage of macular capillaries and the enlargement of the FAZ [67,68]. This is further supported by the inverse correlation of the FAZ and the foveal thickness; individuals with thinner retinas, commonly observed in AD patients who have lower metabolic requirements, end up exhibiting a larger FAZ area [69,70]. The increased size of the FAZ in patients with AD has been supported in vivo by several studies [33,36,37]. However, other studies did not find a significant difference in FAZ measurements between AD patients and healthy controls [34,35,38,39]. Although Chua et al. [38] proved a decrease in vessel density, an increase in the FAZ could not be reproduced. Interestingly, O’Bryhim et al. by designing the only longitudinal study to discover possible OCT-A biomarkers, proved the stable enlargement of the FAZ in the three-year follow-up period in individuals with pre-clinical AD [32,71]. It is worth emphasizing that the FAZ area measurement does not consider the ganglion cell layer, which is known to be affected by neurodegenerative processes [72,73]. Moreover, although enface OCT-A imaging enables the accurate and dependable identification of retinal arteries and veins, ensuring precise vascular mapping, it should be noted that the identification of smaller branches and crossings using OCT-A may be subject to reduced precision and reliability [74].

## 4. Discussion

The heterogeneity of measurement techniques and parameters employed in evaluating vessel density across different retinal areas and layers in various studies has hindered direct comparisons, thus preventing us from drawing a definite conclusion for some possible OCT-A biomarkers in AD [75]. Two different OCT-A systems from Zeiss and Optovue were used in the studies examined. The reproducibility of measurements of VD and the FAZ across different OCT-A systems are not possible, even in healthy subjects [76]. AngioPlex, compared to AngioVue, boasts a shorter execution time, generating a greater quantity of images suitable for analysis while experiencing fewer motion artefacts [77]. Despite its clinical advantage, it has been used in fewer studies. The comparison of studies has been significantly impeded by the utilization of different types of OCT-A machines for measurements, which involve varying algorithms for image reconstruction and the use of different terminologies between Optovue and Zeiss systems, leading to unequal vessel densities between them [44,75]. Enface OCT-A imaging enables the accurate and dependable identification of retinal arteries and veins, ensuring precise vascular mapping. However, as mentioned above, the identification of smaller branches and crossings using OCT-A may be subject to reduced precision and reliability, also contributing to the discrepancy between the studies [74,78]. Another possible limitation is that all the studies included in this analysis employed spectral domain OCT-A, a technique known for its longer imaging duration compared to newer OCT-A models that use swept-source technology [79,80]. Scans obtained from individuals with advanced-stage AD may exhibit artefacts, mainly due to fixation issues during image acquisition. The newer, more technologically advanced swept-source OCT-As, apart from being faster at reproducing images with more details, might replace the older technology, affecting the quality of the biomarkers proposed so far. Moreover, caution should be exercised when interpreting OCT-A findings in this elderly population, as artefacts can compromise the accuracy and reliability of the obtained data. A significant correlation was observed in OCT-A scans between vascular density and the quality factor, indicating that a lower quality factor is associated with lower vascular density, thus affecting the results [42]. Further investigations using alternative OCT-A approaches, utilizing the newer swept-source technology and addressing fixation-related challenges, are warranted to obtain more robust and trustworthy assessments of retinal structures in individuals with advanced-stage AD.

Due to the different segmentation of the retinal layers, even if it was aided in a study by deep learning technology, a direct comparison between the studies was complicated [40]. Researchers utilizing different OCT-A machines should provide comprehensive and precise descriptions of the specific retinal layers being examined, along with the exact criteria used to define their boundaries. Establishing a consensus on the precise boundaries that define regions such as the fovea, parafovea, and perifovea within the macula is crucial; moreover, even if different OCT-A systems are utilized, similar scan area sizes should be adopted by all researchers [31,81]. The studies examined in this review did not use the same scanning area. The absence of standardized protocols for OCT-A image acquisition introduces potential inconsistencies in clinical practice. Therefore, it is crucial to suggest that all future studies should adopt a scan area using the standardized Early Treatment Diabetic Retinopathy Study grid, which sets standard boundaries between the foveal, parafoveal, and perifoveal areas, respectively, as the sector-specific changes that occur throughout the course of dementia may be overlooked when calculations are performed over larger areas [82]. This will allow for a better direct comparison between the studies and allow future investigators to reach safe and reproducible conclusions.

The diversity of participant populations is another probable source of heterogeneity among the included studies. It is well known that both the size of the FAZ and the vessel density have been observed to undergo changes associated with normal ageing and vary among healthy individuals; therefore, these noted changes might also be attributed to this factor [83,84,85]. This was more prominent as certain studies did not match controls and cases based on age. Even in cases where age matching was achieved, it is essential to acknowledge that other potential confounding variables, such as the presence of chronic diseases, were not considered. These uncontrolled factors could introduce bias and influence the associations observed between OCT-A metrics and AD. Although some of the studies, like Chua et al. [38] adjusted for some chronic diseases like diabetes and hypertension, all future studies should consider including comprehensive assessments of participants’ medical histories, including the presence of chronic diseases, to better understand and account for these potential confounders in the analysis. The changes of the superficial vessel density were sex-correlated, with males having a lower vessel density [86]. Therefore, some of the changes reported might be sex-specific, hindering the results’ analysis. Sex and age stratification of the cases and controls is crucial to reduce group variations. Another source of diversity is the fact that the patients in most studies were only clinically diagnosed with AD, thus creating heterogeneous groups with varying levels of tau proteins among them. Only one study in the review required the confirmation of AD through biomarker testing as a diagnostic requirement for participants [36]. The tau level has been associated with a steeper Mini-Mental State Examination (MMSE) decline and a higher risk of AD progression, which might also be reflected in the OCT-A changes [87]. Therefore, the variability in disease severity observed across individual studies could lead to over- or under-estimating the actual effect sizes. It is essential to consider the range of disease severity when interpreting findings, as this variability can impact the magnitude of the associations between OCT-A metrics and AD. By accounting for disease severity as a potential confounding factor, more accurate estimations of the actual effect sizes can be obtained, providing a clearer understanding of the relationships between OCT-A metrics and AD pathology. By including tau levels and MMSE scores and creating specific AD-stratified subgroups, a more focused data analysis can be achieved, overcoming the limitations of small sample sizes in individual studies.

It would be beneficial if the associations of OCT-A metrics with biomarkers derived from other neuroimaging techniques, such as MRI and PET, were explored in order to obtain a thorough grasp of the fundamental neurodegenerative processes in diseases like AD. Additionally, investigating the relationships between OCT-A metrics and biomarkers obtained from other neuroimaging methods can provide valuable insights into the disease pathology and facilitate a more holistic approach in the evaluation of AD. However, the use of the OCT-A as a biomarker for disease progression cannot be established as all the studies except for O’Bryhim et al. [32] were cross-sectional. Cross-sectional studies offer a snapshot of the examined cases at a specific time, providing limited information about the temporal dynamics of retinal vascular changes in AD. Prospective longitudinal evaluations are crucial to a more comprehensive understanding of the relationship between vascular damage and structural alterations in the retina. These large longitudinal studies enable the characterization of longitudinal changes in the retinal architecture and facilitate the examination of their association with cognitive decline. Through such longitudinal follow-up investigations, valuable insights can be gained into the progressive nature of AD and its impact on retinal vascular integrity.

Most of the studies included in this analysis exhibited small sample sizes, which may restrict the applicability of their findings to clinical practice. Although these studies reported statistically significant results, caution is warranted when interpreting the clinical implications and generalizability of the findings. The limited number of participants introduced potential variability and uncertainty in the observed outcomes, making the multifactorial analysis more challenging. Previous research has shown a notable variance in the FAZ area even among normal eyes, being influenced by factors such as gender, central retinal thickness, and retinal vessel density [83,88,89]. These confounding factors underline the importance of conducting studies with larger sample sizes to enhance the precision and reliability of the measurements, thus obtaining more robust and generalizable results [88]. Excluding patients with artefacts in OCT-A scans and those with poor cooperation and with opacities in their ocular media further contributed to the small sample size of the studies reviewed. Therefore, inter-study comparability and further meta-analysis studies, if achievable, might help with addressing this issue and may strengthen their statistical power when analyzing the associations between OCT-A metrics and AD.

It is well established that Alzheimer’s disease predominantly affects elderly individuals [90]. The inclusion of elderly participants in both groups added a layer of complexity to the assessment of the reproduced retinal images. The advanced age of the subjects, a prevalent characteristic within the studies examined, introduced challenges associated with image quality, primarily due to the heightened occurrence of lens opacities, which may potentially lead to less precise measurements of vessel density [91]. Furthermore, it is imperative to discriminate between the decline in vessel density and the expansion of the FAZ, as these processes are inherent to the typical ageing trajectory [63,92]. Age-related reductions in vessel density have a more notable impact on the deep retinal layer compared to the superficial layer [93]. Additionally, age-related macular degeneration, which is more prevalent in the elderly population, can further impact measurements, as it has been reported to reduce vessel density [94]. These age-related changes necessitate careful considerations when interpreting retinal imaging results in older study populations [95].

## 5. Conclusions

Overall, retinal imaging holds promise as a potential noninvasive tool for assessing AD, with the observed alterations in the FAZ area and vessel density suggesting they have potential for being reliable biomarkers. However, further research is needed to validate these findings, establish standardized protocols, and clarify the clinical utility of retinal imaging in AD diagnosis, monitoring, and treatment. Large longitudinal follow-up studies are warranted to better understand the dynamic nature of the FAZ, retinal architecture changes over time, and their association with cognitive decline.

## Figures and Tables

**Table 1:** Segmentation boundaries set by Optovue and Zeiss in the segmentation of the vessel complex.

Device	Optovue	Zeiss
Segmentation for Superficial Vessel Complex	A: 3 µm beneath ILM B: 15 µm beneath IPL	Upper 60% → *ILM up to outer boundary of IPL* [58]
Segmentation for Deep Vessel Complex	A: 15 µm beneath IPL B: 70 µm beneath OPL	Lower 40% → *Inner boundary of IPL up to the outer boundary of OPL* [58]

**Table 2:** A comprehensive overview of the included studies, highlighting key aspects such as the number of participants, the OCT-A device utilized, the scan protocol employed, and the parameters and results investigated within the superficial vessel complex (SVC) and deep vessel complex (DVC). The parameters of interest include the foveal avascular zone (FAZ), vessel density (VD), and direction of observed changes.

Authors	Participants	OCT-A Device	Segmentation Software	Scan Area mm^2^	Parameters	SVC	DVC
O’Bryhim et al. [32]	14 AD 16 control	RTVue XR Avanti	AngioVue	8 × 8	FAZ	FAZ ↑	FAZ ↑
Bulut et al. [33]	26 AD 26 control	RTVue XR 100–2	AngioVue	6 × 6	VD, FAZ	VD ↓ in AD FAZ ↑ in AD	Not analyzed
Yoon et al. [34]	39 AD 133 control	Cirrus HD- 5000	AngioPlex	3 × 3 6 × 6	VD, FAZ	VD ↓ in AD, no significant difference in FAZ	Not analyzed
Lahme et al. [35]	36 AD 38 control	RTVue XR Avanti	AngioVue	3 × 3	VD	VD ↓ in AD, no significant difference in FAZ	No significant difference in VD and FAZ
Zabel et al. [36]	27 AD 27 control	RTVue XR Avanti	AngioVue	6 × 6	VD, FAZ	No significant difference	VD ↓ in AD FA ↑ in AD
Wu et al. [37]	18 AD 21 control	RTVue XR Avanti	AngioVue	6 × 6	VD, FAZ	VD ↓ in AD in one sector FA ↑ in AD	VD ↓ in AD FA ↑ in AD
Chua et al. [38]	24 AD	Cirrus HD- 5000	AngioPlex	3 × 3	VD, FAZ	VD ↓ in AD, no significant difference in FAZ	VD ↓ in AD No significant difference in FAZ
Wang et al. [39]	62 AD 49 control	RTVue XR Avanti	AngioVue	3 × 3	VD, FAZ	VD ↓in AD, no difference in FAZ	No significant difference
Xie et al. [40]	55 AD 62 control	RTVue XR Avanti	AngioVue	3 × 3 6 × 6	VD, FAZ, other microvascular changes (e.g., vascular bifurcations, tortuosity, etc.)	No significant difference	No significant difference
Querques et al. [41]	12 AD 32 control	Cirrus HD- 5000	AngioPlex	3 × 3 6 × 6	Perfusion density	No significant difference	No significant difference
den Haan et al. [42]	48 AD 38 control	Cirrus HD- 5000	AngioPlex	6 × 6	VD, FAZ	No significant difference	No significant difference

↑: Enlargement, ↓: Reduction.

## Data Availability

No new data were created or analyzed in this study.
